# Variability in phenylalanine side chain conformations facilitates broad substrate tolerance of fatty acid binding in cockroach milk proteins

**DOI:** 10.1371/journal.pone.0280009

**Published:** 2023-06-29

**Authors:** Partha Radhakrishnan Santhakumari, KanagaVijayan Dhanabalan, Saniya Virani, Amber S. Hopf-Jannasch, Joshua B. Benoit, Gaurav Chopra, Ramaswamy Subramanian

**Affiliations:** 1 Institute for Stem Cell Science and Regenerative Medicine, Bengaluru, Karnataka, India; 2 Department of Biological Science, Purdue University, West Lafayette, Indiana, United States of America; 3 Manipal Academy of Higher Education, Manipal, Karnataka, India; 4 Department of Chemistry, Purdue University, West Lafayette, Indiana, United States of America; 5 Purdue Institute for Drug Discovery, Purdue University, West Lafayette, Indiana, United States of America; 6 Bindley Biosciences Centre, Purdue University, West Lafayette, Indiana, United States of America; 7 Department of Biological Sciences, University of Cincinnati, Cincinnati, Ohio, United States of America; Pohang University of Science and Technology, REPUBLIC OF KOREA

## Abstract

*Diploptera punctata*, also known as the Pacific beetle cockroach, is a viviparous cockroach that gives birth to live offspring and secretes a highly concentrated mixture of glycosylated proteins as a source of nourishment for developing embryos. These proteins are lipocalins that bind to lipids and crystallize in the gut of the embryo. A structure of milk crystals harvested from the embryos showed that the milk-derived crystals were heterogeneous and made of three proteins (called Lili-Mips). We hypothesized that the isoforms of Lili-Mip would display different affinities for fatty acids due to the ability of the pocket to bind multiple acyl chain lengths. We previously reported the structures of Lili-Mip from crystals grown *in vivo* and recombinantly expressed Lili-Mip2. These structures are similar, and both bind to several fatty acids. This study explores the specificity and affinity of fatty acid binding to recombinantly expressed Lili-Mip 1, 2 & 3. We show that all isoforms can bind to different fatty acids with similar affinities. We also report the thermostability of Lili-Mip is pH dependent, where stability is highest at acidic pH and declines as the pH increases to physiological levels near 7.0. We show that thermostability is an inherent property of the protein, and glycosylation and ligand binding do not change it significantly. Measuring the pH in the embryo’s gut lumen and gut cells suggests that the pH in the gut is acidic and the pH inside the gut cells is closer to neutral pH. In various crystal structures (reported here and previously by us), Phe-98 and Phe-100 occupy multiple conformations in the binding pocket. In our earlier work, we had shown that the loops at the entrance could adapt various conformations to change the size of the binding pocket. Here we show Phe-98 and Phe-100 can reorient to stabilize interactions at the bottom of the cavity–and change the volume of the cavity from 510 Å^3^ to 337 Å^3^. Together they facilitate the binding of fatty acids of different acyl chain lengths.

## 1. Introduction

The ancestors of cockroaches lived as early as 300 million years ago, with their fossils dating back to 120 million years ago [1, 2]. Cockroaches have evolved a reproductive nature that spans oviparous, ovo-viviparous, or viviparous mechanisms [3, 4]. *Diploptera punctata* (Pacific beetle cockroach) is the only known viviparous cockroach that nourishes its young for a considerable period before birth [4]. The mother secretes a concoction rich in proteins, carbohydrates, and lipids called “cockroach milk” to nourish embryos that develop within the brood sac [5–7]. The principal component of this milk is lipocalin-like proteins or Lili-Mips [5–7]. There are 25 cDNA sequences that code for Lili-Mips, which express 22 unique but similar proteins [6, 7]. In the embryo midgut, post-ingestion, Lili-Mip crystallizes [5]. The structure of the crystals showed that at least three Lili-Mip isoforms were present in a single crystal [7]. These sequences, called Lili-Mip1, 2, and 3, are highly expressed during late lactation [8]. These proteins are observed to form *in vivo* crystals despite having heterogeneity in sequence, glycosylation, and bound ligands [5–7].

Lili-Mip has a lipocalin fold and binds to different fatty acids and other moieties [7]. The secondary structure is comprised of an alpha helix and eight antiparallel beta sheets. Lili-Mip is small (18.8 kDa), monomeric, and glycosylated. The calyx, or ligand-binding site, resembles a barrel. Crystal structure and mass spectrometric studies suggest that native and recombinantly expressed Lili-Mips binding pockets contain fatty acids [7, 9].

The remarkable structural similarity of lipocalins warrants the conservation of their unique sequential features. For instance, the overall RMS deviation of Lili-Mip (PDB id 4NYQ) and Human tear Lipocalin (PDB:1XKI) is 4.5 Å for 103 C-alpha atoms. Disulfide bonds and tryptophan at the base of the β-barrel are the most conserved [7, 10–12]. Tryptophan at the N-terminus (Trp-20 in Lili-Mip) covers the base of the 10 Å wide and 15 Å deep ligand-binding calyx [7, 9]. Tryptophan at this position is important for the prevention of retinol oxidation in β-lactoglobulin, for stability in other lipocalins, and plays a role in ligand binding [12, 13]. This single tryptophan makes Lili-Mip, similar to many other members of its family, amenable to binding studies by measuring changes in intrinsic tryptophan fluorescence [13, 14].

**Fig 1:**
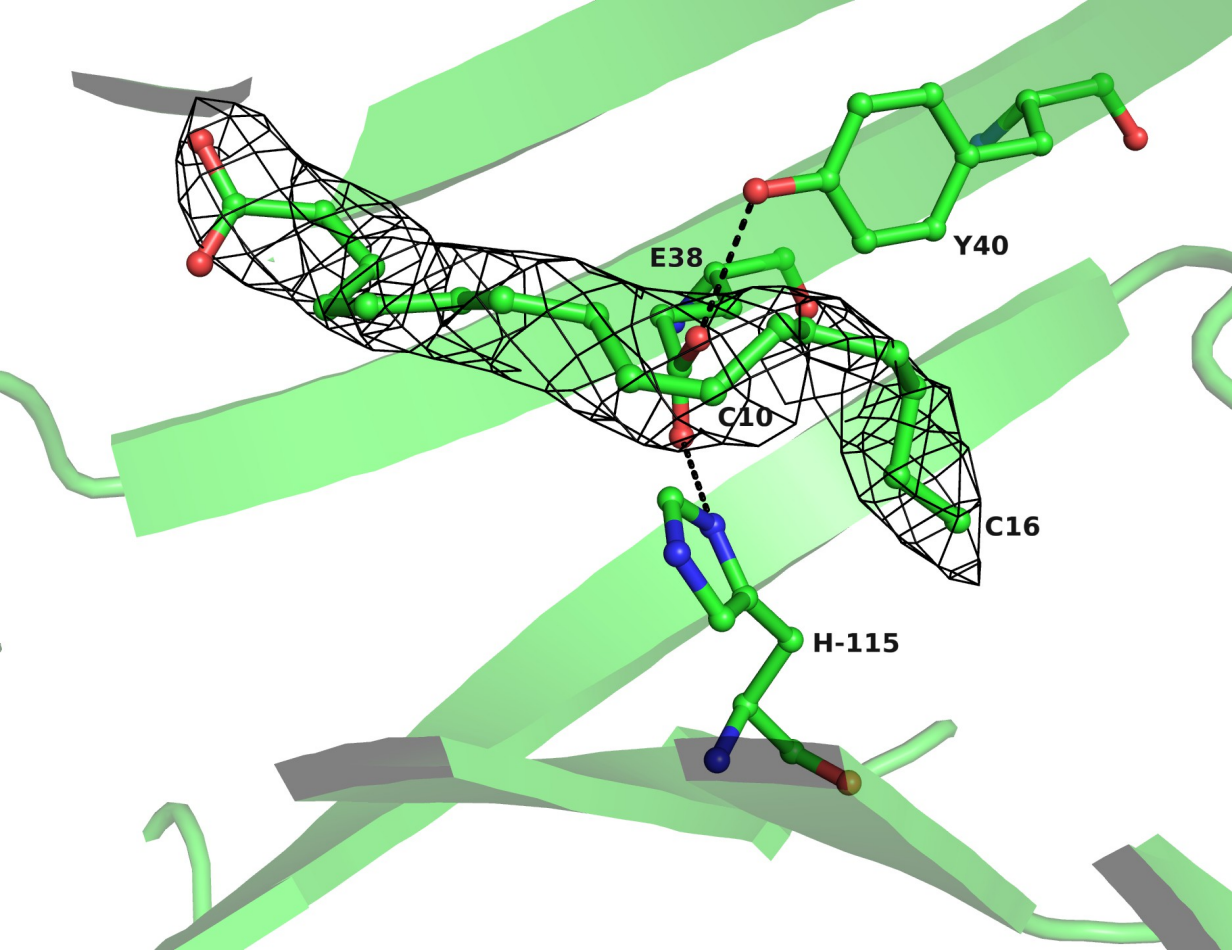
The kink in Lili-Mip2 binding pocket. E38, held in place by Y40 and H115, shapes the cavity near the (ω-7)unsaturated bond of the palmitoleic acid. The figure was made using the deposited structure with PDB-id 7BKX. The (2Fo-Fc) electron density map for the ligand is also shown. The C10 and C16 carbon atoms of palmitoleic acid and the residues are labeled.

The structures of native Lili-Mip and recombinantly expressed Lili-Mip2 provide details on the interaction of fatty acids with residues in the binding pocket [7, 9]. A Glutamate residue (Glu-38) forms a kink in the ligand-binding site of Lili-Mip2, which is held in place by histidine (His-115) and tyrosine (Tyr-40) (Fig 1). The position of the kink from the bottom of the barrel suggests a preference for binding unsaturated fatty acids, with the unsaturated bond positioned at the kink [9]. Fig 1 (made from PDB-id 7BKX) shows the kink at position ω-7 of the modeled palmitoleic acid. When different fatty acids were computationally docked into the protein, like cis-10-hexadecenoic acid, the docking predicted that the unsaturated bond (ω-6) would occupy the position at the kink. In tear lipocalin, charged residues glutamate (Glu-34), histidine (His-84), and lysine (Lys-114) have been shown to interact with the ligand [10]. The same phenomenon was observed for RBP4, a lipocalin-like retinol-binding protein that binds laurate, palmitate, oleate, and linoleate [15, 16]. The carboxylic acid group of the fatty acid is accessible to the solvent, whereas the acyl chain is buried in the hydrophobic interior of the Lili-Mip binding pocket [7, 9].

Previous work in our lab has shown that recombinantly expressed Lili-Mip2 is a thermophilic protein that does not denature even at 95°C at pH 5.0 [9]. Another lipocalin, β-lactoglobulin, is thermodynamically stable in acidic conditions [17]. In this study, we report the stability and binding properties of recombinantly expressed Lili-Mip1, 2, and 3 and compare these factors with the properties of Lili-Mip isolated from the cockroach midgut. We combined the published data on Lili-Mip and other lipocalins with the structural and biochemical data reported here to provide structure-function correlations. These results provide detailed information that can be used to engineer ligand binding by mutating residues in the active site.

## 2. Materials and methods

### 2.1 Cloning

Lili-Mip1, Lili-Mip2, and Lili-Mip3 were synthesized by GeneArt and cloned in pYES2-CT vectors with a C-terminal Histidine tag [7, 9]. Lili-Mip1, 2, and 3 were sub-cloned into the pYES2 vector without an affinity tag. All constructs were codon-optimized for yeast expression and had an N-terminal secretion signal (Ost1 or α-factor) for protein secretion into the medium [18]. A point mutation in Lili-Mip2 at residue 38, from glutamate to alanine (Lili-Mip2-E38A), was generated by site-directed mutagenesis. Lili-Mip2 in the pYES2 vector was modified using forward primer (5’ GTTTCTAACATCACC GCTTTCTACTCTGCTCATG 3’) and reverse primer (5’ GAGCAGAGTAGAA AGC GGTGATGTTAGAAAC 3’) using PCR to generate Lili-Mip2-E38A construct. Sanger sequencing was used to confirm the presence of the mutation.

### 2.2 Expression and purification

*Saccharomyces cerevisiae* strain FGY217 (*MATα*, ura3-52, *lys2_201*, and *pep4*) was used to express and secrete proteins. Yeast was transformed with the Lili-Mip plasmid using the lithium acetate method [19]. Transformed yeast colonies were inoculated in synthetic media lacking uracil with 2% dextrose and grown overnight at 30°C and 220 rpm. The Optical Density (OD) at 600 nm was measured, and the culture was diluted with fresh YPD media to an OD of 0.2/ml. This was again grown until the OD reached 0.6–0.7, induced with 2% galactose, and incubated for 24 h at 30°C and 220 rpm. The supernatant was clarified by centrifugation at 4000 ×g for 10 min. The clarified media was concentrated using a Sartorius Vivaflow200, with a 10kDa molecular weight cutoff filter to a final volume of 500 ml. The media was buffer exchanged into 50 mM sodium acetate at pH 5.0 and 10 mM NaCl while concentrating the media. The buffer-exchanged media were purified using cation exchange chromatography on an SP FF column (Cytiva). The Lili-Mip protein eluted between 100 mM and 200 mM NaCl (on a gradient that extended to 1.0M NaCl). The protein fractions were concentrated using a 10 kDa filter in an Amicon spin filtration system. The concentrated protein was purified using the size exclusion chromatography column S75 (Prep Grade—Cytiva) in 50 mM Sodium acetate pH 5.0 and 50 mM NaCl buffer.

### 2.3 Lipid extraction and delipidation of Lili-Mip

Lili-Mip was delipidated using the Bligh Dyer method [20]. The chloroform phase containing the fatty acids was dried and used for fatty acid analysis by LC-MS/MS. The aqueous phase containing the delipidated protein was dried and resuspended in an appropriate buffer for the binding studies.

### 2.4 Intrinsic tryptophan fluorescence

Fluorescence measurements were carried out in 0.05 M sodium acetate buffer pH 4.8 or 0.05 M sodium phosphate buffer pH 4.8/7.2/8.2, containing 100 mM NaCl buffer. Delipidated Lili-Mip1, 2, or 3 were diluted to 4 μM in the buffer, and ligand (dissolved in ethanol) was added in multiple concentrations between 0–35 μM. Protein solutions were incubated for 1 min at room temperature (25°C). The samples were excited at 290 nm (slit 2.5 nm), and the emission was measured at 325 nm (slit 2.5 nm) using a Cary fluorescence spectrophotometer. The data analysis was performed as described by Weise et al. [21].

### 2.5 Targeted analysis of fatty acids by LC/MS/MS

An Agilent 1290 Rapid Resolution liquid chromatography (LC) system coupled to an Agilent 6470 series QQQ mass spectrometer (MS/MS) was used to analyze the fatty acids in each sample (Agilent Technologies, Santa Clara, CA, USA). The methods used in this study were similar to those described by Yang et al., with some minor modifications [22]. The internal standard for the assay was D_3_-CMP derivatized pure fatty acids. Each sample was spiked with 50 ng of internal standard before analysis. An Agilent Eclipse Plus C-18 (2.1 mm x 50 mm) 1.8 μm column was used for LC separation. The buffers were (A) water + 0.1% formic acid and (B) isopropanol/acetonitrile (50/50 v/v) + 0.1% formic acid. A linear LC gradient between 10 and 100% B was used. The post time was set to 6 min. The flow rate was 0.3 mL/min. Multiple reaction monitoring was used for the MS analysis. The data were acquired in positive electrospray ionization (ESI) mode. The jet stream ESI interface had a gas temperature of 325°C, a gas flow rate of 8 L/minute, a nebulizer pressure of 45 psi, a sheath gas temperature of 250°C, a sheath gas flow rate of 7 L/minute, a capillary voltage of 4000 V in positive mode, and nozzle voltage of 1000 V. The ΔEMV voltage was 300 V. Agilent Mass Hunter Quantitative analysis software was used for data analysis (version 10.1).

Native gut crystals were collected using the methods described previously by Banerjee et al. [7]. The cockroaches were reared at the University of Cincinnati (UC) in a climate-controlled facility. The cockroaches used in this study were of the same species as those used in previous milk protein studies [6] and genome sequencing [23]. The ambient temperature was maintained between 24 and 28°C, and the relative humidity (RH) was held between 70% and 80%. A 12:12-hr light-dark photoperiod was maintained for the duration of the experiment. Milk protein crystals were isolated from embryos late in pregnancy [8]. Native Lili-Mip crystals were extracted in 50% PEG400, and the samples were shipped in 50% PEG400. The crystals were dissolved in appropriate buffers for biochemical studies.

### 2.6 pH probing in the gut

The pH levels were assessed using a Thermo Scientific Orion micro-pH probe based on a protocol adapted for cockroaches from tsetse flies [24]. Specifically, females were dissected to remove embryos during the late stages of lactation. The embryos were dissected to remove the guts. The guts were ruptured and centrifuged through a fine mesh (polyester, 150 mesh) filter at 2,000 xg for 2 min to separate the gut contents from the gut. The pH of the contents of the gut lumen (containing Lili-Mips), cells of the gut (homogenized), and remaining body parts (homogenized) were determined.

### 2.7 Docking calculations

CANDOCK (version 0.6.0), an in-house docking software, was used to generate docking conformations of all fatty acids with Lili-Mip2 (PDB ID: 7Q02), including a selection of the binding site [25]. The fatty acid structures of myristic acid (14:0), palmitoleic acid (16:1), and linoleic acid (18:2) were drawn on BioChemDraw, cleaned in 3D, and saved as Mol2 files for docking. CANDOCK was used with default parameters, and the radial-mean-reduced-6 (RMR6) was used as the “Selector” parameter for docking to select the top pose as benchmarked previously [25]. The docking scores of all 96 potential energy functions were calculated for all docking states. The protonation states of the protein bound to the fatty acids were determined at four pH values: 4.7, 5.9, 7.2, and 9.1. The distance from the last carbon atom on the fatty acid to carbon CH_2_ on Trp-20 in the binding pocket was calculated using VMD software for each docked pose, and the fatty acid carbon atoms, C14 on myristic acid, C16:1 on palmitoleic acid, and C18:2 on linoleic acid were used [26].

### 2.8 Molecular dynamics simulations

The docked pose of the fatty acid with the lowest distance (3–5 Å) from carbon CH_2_ on W20 of Lili-Mip2 was used for Molecular Dynamics (MD) simulations with GROMACS [27]. MD calculations were performed for 200 ns with the docked conformation of each fatty acid with the protein surrounded in explicit water molecules. We used the CHARMM-26 force field and TIP3P water models in a 42 Å cubic box with periodic boundary conditions [28, 29]. To prepare the system for MD, we ran 50,000 steps of steepest descent energy minimization, with an initial step size of 0.1 Å. The particle mesh Ewald (PME) scheme was used for long-range electrostatics [30], using a Coulomb cutoff distance of 1.2 Å. A van der Waals (VdW) cutoff type was specified with a cutoff distance of 1.2 Å. All other parameters in energy minimization were set to default values in GROMACS. After energy minimization, a 100 ps MD simulation was run in the NVT ensemble (constant number of molecules, volume, and temperature) using the Berendsen thermostat at a reference temperature of 300 K, sampled at every 0.1 ps with a chain length of 4 for equilibration. The electrostatic cutoff distance was set to 1.5 nm. To obtain the isothermal-isobaric NPT ensemble (constant number of molecules, pressure, and temperature), we used a time step of 0.002 ps with a Berendsen thermostat at a reference temperature of 298 K and a time constant of 1 ps for the entire system and the Berendsen barostat [31] at a reference pressure of 1 bar with a time constant of 2 ps and a compressibility value of 4.5e-5 bar^-1^. A final production MD simulation was done for 200 ns with initial velocities generated by a Maxwell distribution at 298 K. The fatty acid was not constrained during the equilibration and production MD run. All other parameters in the MD run were set to default values used by GROMACS.

### 2.9 Deglycosylation using PNGase

Recombinant Lili-Mips were treated with PNGase F (New England Biolabs) to remove N-linked oligosaccharides. Glycosylated Lili-Mip and PNGase F were mixed in a buffer having 1X GlycoBuffer-2 (0.05 M Sodium phosphate pH 7.0, 0.2 M EDTA). The reaction mixture was then incubated at 37°C for 24 h. SDS-PAGE was used to differentiate between glycosylated and deglycosylated Lili-Mip proteins.

### 2.10 Crystallization

The Lili-Mips were concentrated using 10kDa Amicon filters. The calculated extinction coefficient of 28100 M^-1^ cm^-1^ was used to measure the concentration using a NanoDrop 2000 spectrometer (Thermo Fisher). Hanging drops were set up using a Mosquito robot (200nl protein + 200nl well solution with a 50ul well solution). Lili-Mip2-E38A crystals were obtained in 0.002 M Zinc sulfate heptahydrate, 0.08 M HEPES pH 7.0; 25% v/v Jeffamine® ED-2003 as the precipitant at 20°C. Lili-Mip1 crystals were obtained in 0.2 M Potassium Nitrate pH 6.9; the precipitant was 20% (W/V) PEG 3350. The crystals were cryoprotected by the addition of 5% glycerol.

### 2.11 Data collection, structure solution, and refinement

The data for the Lili-Mip2-E38A mutant were collected at the MBC-CAT beamline at the Advanced Light Source at Berkeley (900 frames at a 0.2-degree oscillation). The data were processed using XDS [32] and scaled using the Aimless package in the CCP4-suite [33, 34]. The data were good to 2.95 Å resolution. The structure was determined by molecular replacement using the deposited structure of Lili-Mip (PDB ID 7BKX) as a model. The maps clearly showed the absence of a side chain at position 38. The structure was refined using Refmac to a final R-factor and R-free of 23.4 and 29.4, respectively [35]. Data collection, refinement, and structure quality statistics are presented in S1 Table. The data for Lili-Mip1 were collected at the APS GMCA-CAT beamline ID 23D-D. Data processing with XDS and scaling suggested good data to 2.16 Å resolution. The structure was determined by molecular replacement using the deposited 4NYQ model. The refined model had a final R-factor and R-free of 0.19 and 0.25, respectively. For both structures, iterative model building was carried out with the program Coot [36]. Model figures and plots were made with Pymol [37] and R [38].

### 2.12 Thermal stability assay

Tycho NT.6 (Nano Temper) was used to measure the melting temperature (T_m_) of recombinant Lili-Mips and proteins with mutations. Protein stability was measured in different buffers (50 mM HEPES 7.4 and 50 mM sodium phosphate buffer ranging from pH 4.8 to 9.3, with NaCl concentrations ranging from 0.5 M to 2 M) to determine the pH and salt dependence of stability. Protein concentration was set to 0.1mg ml^-1^. The heating range was between 35°C—95°C.

## 3. Results and discussion

**Fig 2:**
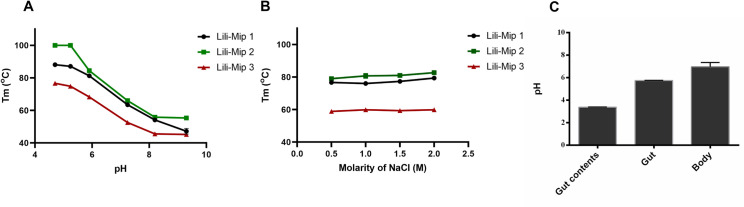
Temperature dependent denaturation of recombinant Lili-Mip 1, 2 and 3 with respect to pH and salt. (A) Left -Melting temperature (T_m_) of Lili-Mip1, 2 and 3 in 50mM sodium phosphate buffer at pH 4.8, 5.2, 5.9, 7.2, 8.2, 9.3 as mentioned in methods. (B) Melting temperature (T_m_) of Lili-Mip1, 2 and 3 in 50mM HEPES pH 7.4 at different NaCl concentrations—0.5M, 1M, 1.5M and 2M. Data was collected on TychoNT.6 (Nanotemper). The experiments are repeated with three biological replicates, n = 3 and are represented as mean ± S.D. (C) pH of the gut contents (primarily Cockroach milk), cells of the gut and body measured using a microprobe shows Lili-Mip is stored in acidic conditions whereas the cells are at a higher pH. The experiments are repeated with two cockroaches(n = 2) and are represented as mean ± S.D.

Lili-Mip1, 2, and 3 are thermophilic proteins at low pH (4.8). Lili-Mip2 is the most thermostable, followed by Lili-Mip1 and 3. Upon increasing the pH, the stability of Lili-Mip1, 2, and 3 markedly decreased. Lili-Mip2 had the maximum melting temperature (ΔT_m_) change of around 50°C on pH change from 4.8 to 9.3 (Fig 2A). The thermostability was independent of the ionic strength (0.5M to 2M NaCl), indicating that the Lili-Mip1, 2, and 3 are also stable at high salt (Fig 2B). Although Lipocalins are known to be thermostable, such a large change in T_m_ has not yet been observed. We tested a few other lipocalins to check if thermal stability changed with pH and if this phenomenon is a property of the lipocalin fold. Neither cellular retinoic acid-binding protein (CRABP1) nor Sandercyanin Fluorescent protein (SFP) showed a change in thermostability with variation in pH (S1 Fig). However, it has been shown that the thermostability of β-lactoglobulin is pH dependent [17]. Deglycosylated Lili-Mip1 showed a marginal decrease in stability (S2 Fig). However, the removal of glycans did not affect the pH-dependent change in the thermal stability of Lili-Mip1. The effect of delipidation on the thermostability of Lili-Mip1, 2, and 3 was investigated. Delipidated Lili-Mip isoforms show the same pH-dependent changes in thermostability (S3 Fig). However, the difference in thermal stability was not significant. We concluded that pH-dependent changes in the thermal stability of Lili-Mip isoforms are a property of the protein, and neither glycosylation nor fatty acid binding play a role.

To understand the physiological relevance of this, we measured the pH of the embryo midgut and the cells of the embryo. We show that the gut contents of the cockroach milk containing Lili-Mips have an acidic pH of 3.38. However, the gut cells had a pH of 5.76. The remaining body showed a pH of 6.96 (Fig 2C). Gut cells are maintained in a more basic environment than the gut lumen, where the milk proteins reside. This provides a possible explanation for this behavior.

The embryo ingests Lili-Mip into its midgut, where it is stored until required. The increased stability of Lili-Mips at acidic pH enables the long-term storage of the protein and its ligand (fatty acid). Lili-Mips can be taken into the cells when the embryos need food and assimilated. We hypothesize that the decreased stability in higher pH makes it easier to be broken down and used as food. This hypothesis needs further experimental validation by identifying the organelles in the gut cells where the protein is endocytosed and pH measurement inside these organelles. Still, the pH gradient identified suggests that increasing pH is likely a critical factor for the ease of breakdown of the milk protein crystals.

**Fig 3:**
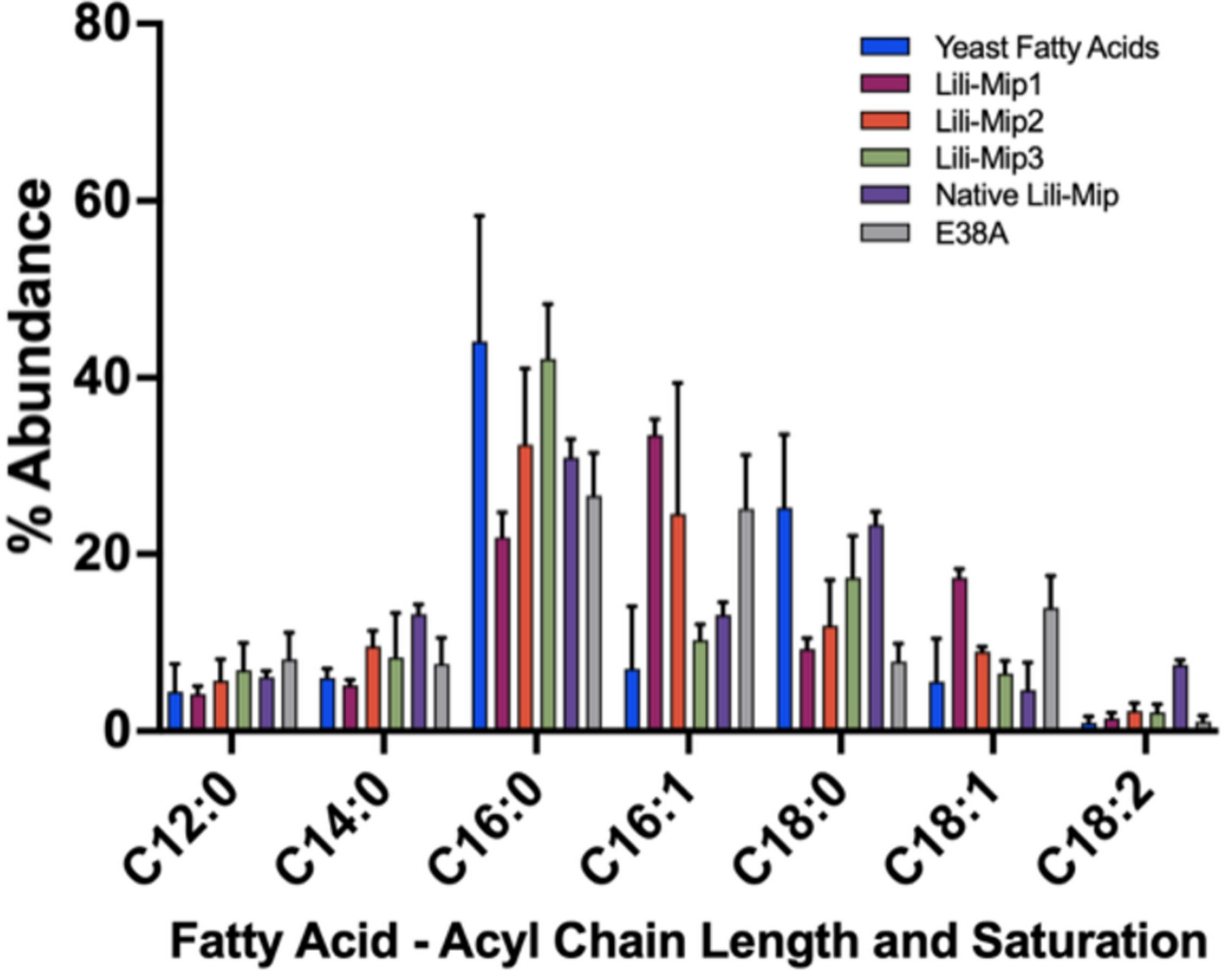
Fatty acid profile of recombinant and native Lili-Mips using LC/MS/MS. (A) Fatty acids bound to Lili-Mip1, 2, 3, Lili-Mip 2 with E38A mutation, and native Lili-Mip crystals. Each fatty acid peak density was compared with the corresponding standard. The experiments are repeated with three biological replicates, n = 3 and are drawn as mean ± S.D.

Mass spectrometry analysis showed that all Lili-Mip isoforms (and Lili-Mip2-E38A) bound abundantly to palmitoleic acid (16:1), palmitic acid (16:0), oleic acid (18:1), and stearic acid (18:0). Similar experiments showed that palmitic acid (16:0), stearic acid (18:0), palmitoleic acid (16:1), and linoleic acid (18:2) were the most abundant fatty acids bound to the native Lili-Mips (Fig 3). We hypothesized that the kink formed by E38 in Lili-Mips results in a preference for the binding of unsaturated fatty acids, with the unsaturated bond occupying the position at the kink. Contrary to our hypothesis, there was no preferential binding to saturated versus unsaturated fatty acids. Notably, 16- and 18-carbon fatty acids are the predominant fatty acids in yeast. Native Lili-Mip also follows the trend, the only difference being the increased presence of linoleic acid. This could be attributed to the fact that linoleic acid is present in *Diploptera punctata* but not in *Saccharomyces cerevisiae* [7]. These results suggest that the abundance of fatty acids bound to Lili-Mip depends on the endogenous composition of fatty acids. In other words, Lili-Mip1, 2, and 3 show similar specificity to fatty acids (Fig 3). The differences observed in Fig 3 were not statistically significant. The ability of the Lili-Mip isoforms to bind to fatty acids of different acyl chain lengths provides the embryo with a variety of nutritional contents required for its growth and development [39, 40].

We showed that native or recombinant Lili-Mips could bind to different fatty acids. We exploited a single tryptophan (Trp-20) at the base of the ligand-binding site to measure the binding affinity of fatty acids to Lili-Mip1, 2, and 3. The fluorescence intensity of tryptophan increases upon ligand binding. The intrinsic tryptophan fluorescence change was employed to determine the binding affinity of different fatty acids to Lili-Mip1 (S4 Fig). Fatty acids of varying chain lengths (14, 16, and 18) and degrees of unsaturation (0, 1, and 2) were assayed. All tested fatty acids bound with micromolar range affinities to the different Lili-Mips (Table 1). These data suggest that the acyl chain length or the presence of double bonds does not significantly affect binding affinity. This implies that the fatty acids the embryo receives almost exclusively depend on the fatty acid composition of milk-secreting cells within the brood sac.

**Table 1:** Binding affinity of Lili-Mip1, 2 and 3 with different fatty acids. Intrinsic Tryptophan fluorescence intensity change is employed to measure the binding affinities of Lili-Mip1, 2 and 3 for different fatty acids. All values are in μM. The protein is in 50mM sodium acetate buffer pH 4.8, 100mM NaCl. The experiments are repeated with three biological replicates, n = 3 and are drawn as mean ± S.D.

K_D_ in μM	Myristic acid (14:0)	Myristoleic acid (14:1)	Palmitic acid (16:0)	Palmitoleic acid (16:1)	Stearic acid (18:0)	Oleic acid (18:1)	Linoleic acid (18:2)
Lili-Mip1	1.96±0.83	2.06±0.42	1.76±0.88	1.54±0.91	1.71±0.56	0.93±0.31	1.56±0.33
Lili-Mip2	2.44±2.68	2.78±0.50	1.41±0.88	2.30±1.32	2.32±1.32	1.71±1.19	2.19±1.41
Lili-Mip3	1.60±0.51	0.68±0.49	0.82±0.61	0.88±0.62	1.78±1.74	0.24±0.11	0.27±0.08

Binding studies with Lili-Mip1 against palmitic acid were performed at different pH values to verify whether the fatty acid binding affinity was pH-dependent. The data showed that Lili-Mip1 had no change in binding affinity for palmitic acid between pH 4.8 and 8.2(K_D_’s at pH 4.8, 7.2, and 8.2 for Palmitic acid are 1.76± 0.93; 0.44±0.20; 2.24±0.11 μM respectively). Lili-Mip2-E38A did not exhibit fluorescence intensity change on ligand binding. Therefore, we could not measure ligand binding affinity even though mass spectrometry analysis showed fatty acid binding (Fig 3). The crystal structure of Lili-Mip2-E38A suggested that the binding cavity did not extend to tryptophan (S5A and S5B Fig). In the bound fatty acid in the structure, the distance between the terminal methyl group of the acyl chain of the fatty acid and the ring of the Trp is 8.4 Å, compared to 3.8 Å in Lili-Mip 2. We propose that binding of the fatty acid at that distance will not significantly change the tryptophan environment resulting in a change in intrinsic tryptophan fluorescence (S5C and S5D Fig).

The structures of Lili-Mip1(PDB ID: 8F0Y) and Lili-Mip2-E38A (PDB ID: 8F0V) showed a density for the bound fatty acid. However, it is difficult to ascertain the length and nature of the bound fatty acid. The structure of Lili-Mip2 showed that in the ligand-binding pocket, Glu-38 is held in position by hydrogen bonds from His-115 and Tyr-40, enabling ligand binding [9]. The mutation of Glu-38 to alanine was predicted to influence ligand binding. Mutation to alanine only slightly altered the positions of Tyr-40 and His-115. Furthermore, biochemical data indicate that these proteins bind to multiple fatty acids (Fig 3). This correlates with the lack of clear density in the crystallographic data suggesting heterogeneity in fatty acid binding among the molecules that constitute the lattice.

**Fig 4:**
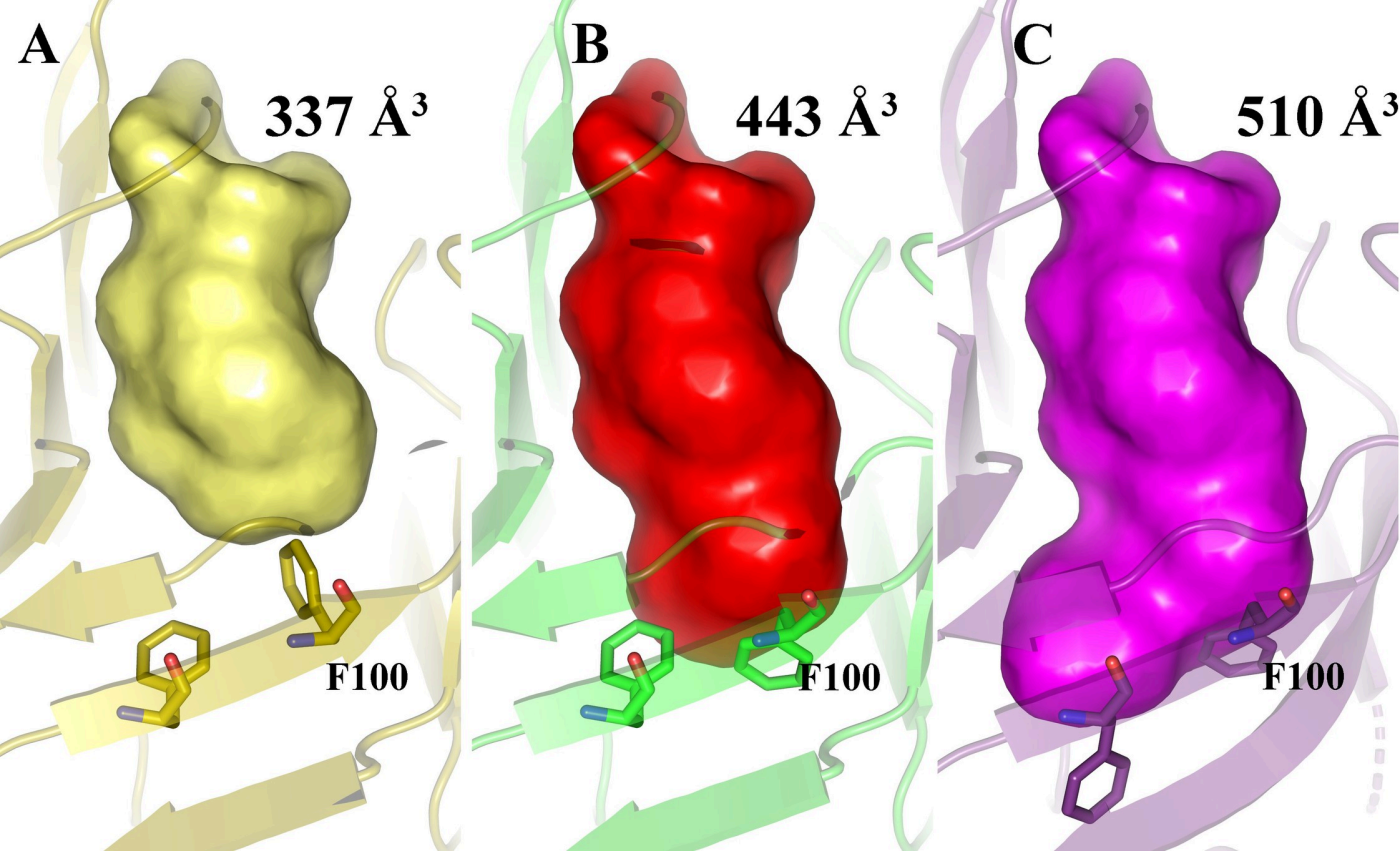
Cavity volumes of Lili-Mip with Phe-98 and Phe-100 in different conformations. Phe-98 and Phe-100 were modeled in different conformations observed on the structure of Lili-Mip (PDB id 4NYQ). Parkvfinder was used to find cavity volumes(surface) in Lili-Mips in order to see the effect of F98 and F100 on ligand binding. (A) The volume of the cavity when both Phe-98 and Phe-100 point towards the entrance (yellow). The volume of the cavity is 337 Å^3^. (B) the Volume of the cavity when Phe-98 points towards the calyx and Phe-100 points towards the botton (red). The volume of the cavity is 443 Å^3^. (C) The volume of the cavity when Phe-98 and Phe-100 point towards the bottom of the cavity (magenta). The volume of the cavity is 510 Å^3^. is when the side chain of the Phe points towards the entrance, down when it points towards the bottom of the barrel.

In the Lili-Mip1 structure, we found that Phe-98 and Phe-100 moved toward the entrance of the binding pocket compared to Lili-Mip2, where these residues pointed toward the bottom of the binding pocket, causing a decrease in the size of the Lili-Mip2 (PDB id 7BKX) ligand-binding pocket relative to Lili-Mip1. In the structure of the in vivo grown crystals (PDB id 4NYQ), Phe-100 had partial occupancy–one conformation pointed down to the bottom of the cavity and another towards the calyx (S6 Fig). These observations suggested that Phe-100 and Phe-98 could occupy multiple conformations to stabilize the binding of fatty acids of varying acyl chain lengths. To better understand how the ligand-binding pocket size changes between structures, we quantified the cavity volumes (using the parKVfinder software [41]) of Lili-Mip (4NYQ), where we modeled the Phe-98 and Phe-100 in the different conformations using coot [36]. Fig 4 shows the different orientations of Phe-98 and Phe-100 observed in the structures. The decrease in the binding pocket size directly correlates with the side-chain orientation of Phe-98 and Phe-100. When both Phe-98 and Phe-100 point towards the entrance of the binding pocket, the volume is lowest at 337 Å^3^ (Fig 4A). When Phe 100 changes conformation to point towards the bottom of the cavity, and Phe-98 points towards the entrance, we get an intermediate volume of 443 Å^3^ (Fig 4B). When both point towards the cavity’s bottom, the volume increases to 510 Å^3^ (Fig 4C). Interestingly when Phe-100 points towards the entrance, the volume of the cavity is the same as both point towards the entrance, as it blocks further access to the bottom of the cavity.

We conclude that Phe-98 and Phe-100 have a bearing on cavity volume. The different conformations of Phe-98 and Phe-100 allow Lili-Mips to bind fatty acids of different lengths, thereby explaining the broad substrate tolerance of Lili-Mips. The thermodynamics of binding that stabilizes a specific acyl chain length in the binding pocket is in equilibrium with the kinetic process. This is supported by the results shown in Fig 3, where all proteins are bound to fatty acids with multiple acyl chain lengths. Whether the conformational preference of these residues determines fatty acid binding or fatty acid binding induces a conformational change to stabilize the bound fatty acid is hard to predict. Importantly, in our earlier work, we have also shown that the loops at the entrance are also flexible [7]. Notably, in the manuscript by KanagaVijayan et al., in Fig 3E, we show evidence that while two different fatty acids could bind to the same depth, their head groups can occupy different positions [9]. These changes in loop structures can also affect the cavity’s overall volume and stabilize the headgroup’s binding.

**Fig 5:**
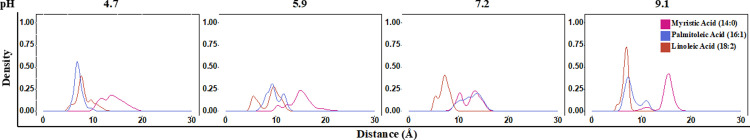
Molecular simulations of Lili-Mip 2(PDB ID: 7Q02) shows nature of fatty acid binding. Probability density distance plots myristic acid (pink), palmitoleic acid (blue) and linoleic acid (orange) at different pH.

MD simulations were performed to understand Lili-Mip2’s ligand binding properties at differing pHs (4.7–9.1). We calculated the distance between Trp-20 and the terminal carbon of the fatty acid to track the location of fatty acid over a 200-ns period. Fig 5 shows the probability density for the distance of the fatty acid from Trp-20 and is a proxy for the binding depth of the fatty acid. At pH 4.7, the peak for palmitoleic acid was approximately 5 Å, suggesting that it was fully buried 50% of the time. However, at pH 5.9, the peak was longer and shallower, implying a lower population of well-bound palmitoleic acid. The probability density plot for fatty acid binding in Lili-Mip2 showed that at a pH of 4.7, the fatty acid stayed in the ligand-binding pocket longer than at the pH inside the cells (Fig 5). This result, along with the lower stability with increasing pH, supports the idea that once absorbed into the cells, the protein can be broken down, and fatty acids, amino acids, and sugars can be used for embryo growth and development. While linoleic acid and palmitoleic acid show a similar trend going from pH 4.7 to pH 5.9, as the pH continues to increase, linoleic acid seems to bind deeper into the binding pocket. The physiological relevance of this is hard to interpret with our current data. Myristic acid binds farther from Trp-20 than palmitoleic acid because of its shorter chain length. The broad peak indicated multiple binding modes inside the pocket (pocket larger than the acyl chain length). This resulted in similar stabilization energies across the modes. Similar to that observed for other lipocalins, the simulations also suggest that the residues that change conformation the most are at the entrance of the binding pocket (S7 Fig). Interestingly as the pH increased to 9.1, the probability density plots suggested that the binding was tighter. However, the physiological impact of this observation remains unclear.

In summary, results from our studies on the fatty acid binding profile, binding affinity, crystal structures of Lili-Mip1, and molecular dynamics simulations showed that Lili-Mips bind with similar affinity to several fatty acids. This provides a variety of fatty acids required by the embryo as nutrients for growth and development. Lili-Mips are highly thermostable proteins, and their stability is pH dependent. We hypothesized that this allows the protein-lipid-glycan complex to be very stable in the gut and less stable inside the cell, where the pH is close to neutral. Measurements of pH in the gut and cells confirmed the differences in pH between gut contents and the gut cells. We further propose that this difference in pH and the corresponding changes in stability between the embryo’s gut and inside the cells changes the folding kinetics, making it easier for proteases to break them down. This would allow the cells to break down Lili-Mips and use them as a source of metabolites ‐ amino acids, fatty acids, and sugars–to promote embryo growth within the brood sac. This hypothesis requires physiological validation, which would require experimental determination of the mechanism of uptake and the physiological state of the organelles within the cells where the proteins are digested. The Lili-Mips are highly thermostable. The thermostability does not change much on deglycosylation or ligand binding, suggesting that it is an intrinsic property of the protein structure. The changes in the conformation of the Phe-98 and Phe-100, combined with the ability of the loops at the entrance to adopt different conformations, seem to provide Lili-Mips the ability to bind fatty acids of various lengths. The different orientations of Phe-98 and Phe-100 help stabilize the binding of fatty acids with different acyl chain lengths to bind with similar affinities. Structural work with different acyl chain fatty acids binding to Lili-Mips one at a time would be required to test this interpretation.

## Supporting information

S1 Fig Comparison of thermal stability of different lipocalins with change in pH. Sandercyanin Fluorescent Protein (SFP) and cellular retinoic acid binding protein 1(CRABP1) are lipocalins that are structurally like Lili-Mip-2. Yet they do not show a significant change in thermal stability as compared to Lili-Mip-2. The experiments are repeated with three biological replicates, n = 3, and are drawn as mean ± S.D.
(TIF)

S2 Fig Thermal stability of Lili-Mip-1 and deglycosylated Lili-Mip-1. Glycosylation adds to the stability of Lili-Mip across pH. However, the decrease in stability of Lili-Mip-1 with pH is not primarily due to glycans. The experiments are repeated with three biological replicates, n = 3, and are drawn as mean ± S.D.
(TIF)

S3 Fig Comparison of thermal stability of recombinant and delipidated Lili-Mip-1, 2, and 3 with respect to pH. Thermal denaturation of Lili-Mip-1, 2, and 3 and delipidated ones show a marginal decrease in stability, more pronounced in acidic pH in Tycho. The experiments are repeated with three biological replicates, n = 3, and are drawn as mean ± S.D.
(TIF)

S4 Fig Ligand binding curve for Lili-Mip 1 with myristic acid. Representative figure showing the titration of Lili-Mip-1 with 0μM to 35μM myristic acid. The experiments are repeated three times (three biological replicates).
(TIF)

S5 Fig (A) shows the cavity of Lili-Mip2 in green. E38, as in the main paper Fig 1, shows a kink. E38 and the Tryptophan residue are marked. (B) The cavity of LiliMip 2 with E38A mutation–shows that the cavity does not extend close to the TRP. (C) The bound fatty acid in LiliMip 2 –the distance between the terminal methyl group and the indole ring of the tryptophan is 3.8Å. (D) The bound fatty acid in LiliMip 2 with E38 mutated to A. The distance between the terminal methyl group and the indole ring of tryptophan is 8.4Å. Cavity figures were made with the software CavityPlus [42]
(TIF)

S6 Fig Phenylalanine 100 occupies two conformations with partial occupancies in the structure determined from *in vivo* crystals. This demonstrates the flexibility of the side chain. The diagram was made by downloading the 4NYQ coordinates and 2Fo-Fc maps from the protein data bank.
(TIF)

S7 Fig Analysis of Lili-Mip 2 bound to palmitoleic acid via docking and molecular dynamic simulation. (A) Distance of W20 on LiliMip 2 and C16:1 on palmitoleic acid of docked conformation. (B) The final conformation of LiliMip 2 (green) from the MD simulation is superimposed on its crystal structure (grey).
(TIF)

S1 Table Data collection and refinement statistics for Lili-Mip-2-E38A and Lili-Mip-1. (DOCX)

S1 Data Supplementary tabular data used to make Fig 3. (XLSX)
