# Supplementary figures and images for: Variability in phenylalanine side chain conformations facilitates broad substrate tolerance of fatty acid binding in cockroach milk proteins
Source: PLoS One. 2023 Jun 29;18(6):e0280009. doi: 10.1371/journal.pone.0280009 (PMC10310036; doi:10.1371/journal.pone.0280009)

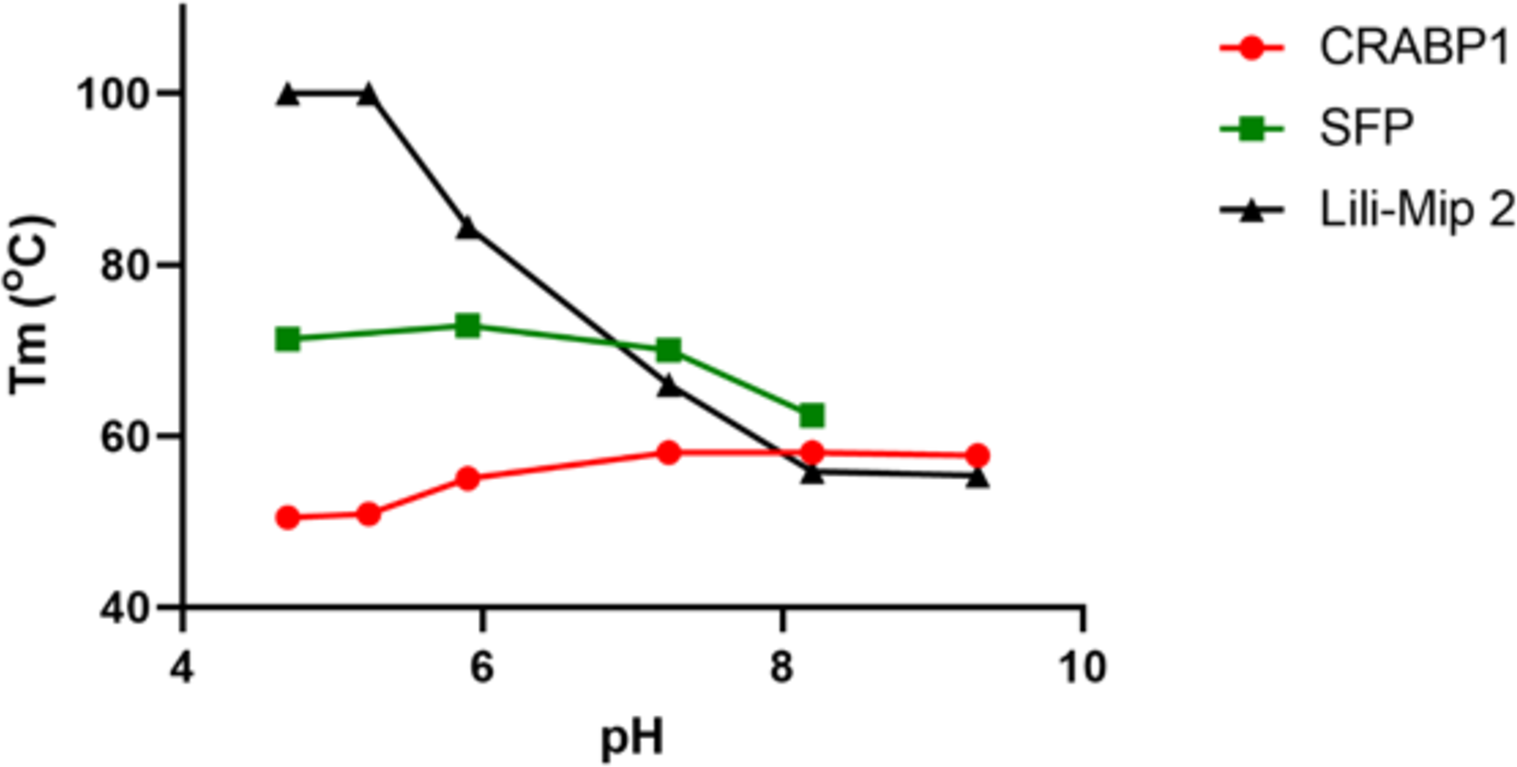

Supplement: S1 Fig — Sandercyanin Fluorescent Protein (SFP) and cellular retinoic acid binding protein 1(CRABP1) are lipocalins that are structurally like Lili-Mip-2. Yet they do not show a significant change in thermal stability as compared to Lili-Mip-2. The experiments are repeated with three biological replicates, n = 3, and are drawn as mean ± S.D. (TIF) [file pone.0280009.s001.tif]

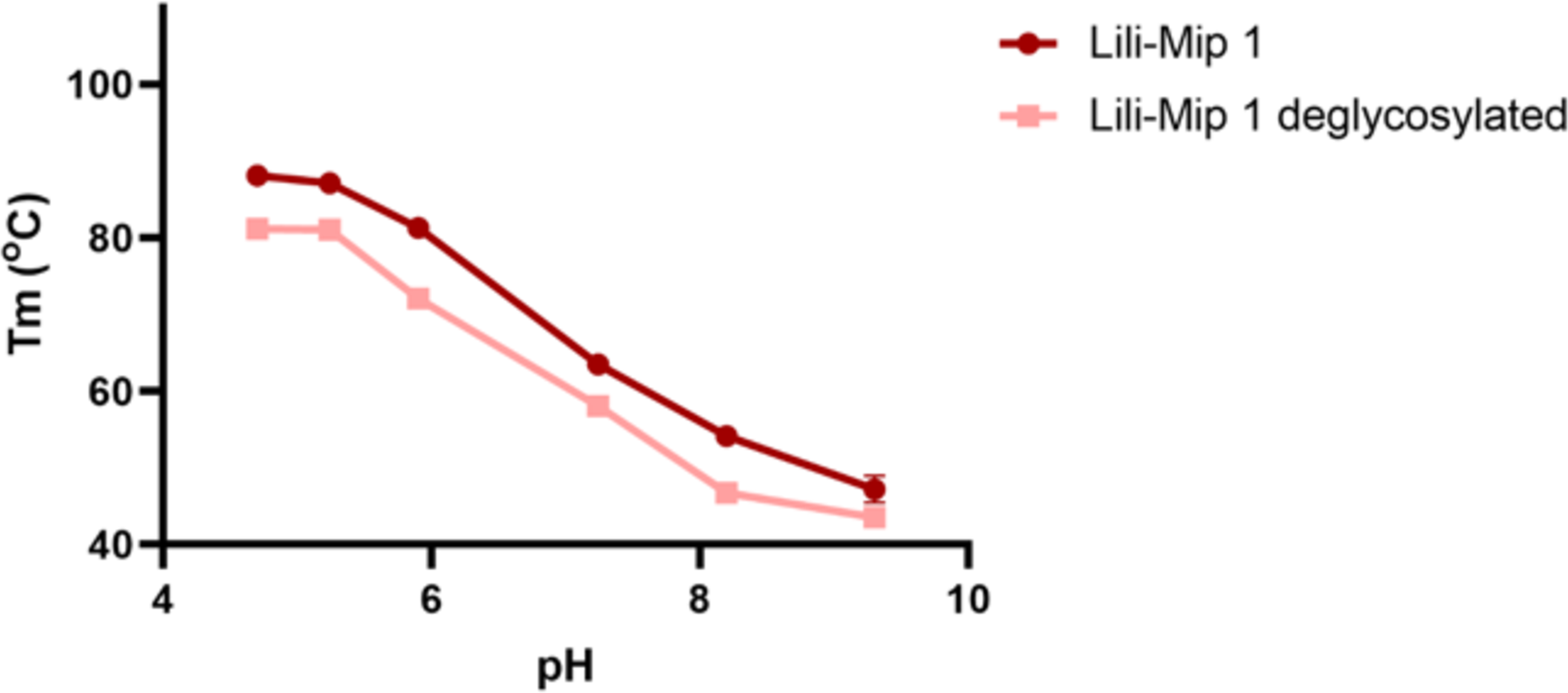

Supplement: S2 Fig — Glycosylation adds to the stability of Lili-Mip across pH. However, the decrease in stability of Lili-Mip-1 with pH is not primarily due to glycans. The experiments are repeated with three biological replicates, n = 3, and are drawn as mean ± S.D. (TIF) [file pone.0280009.s002.tif]

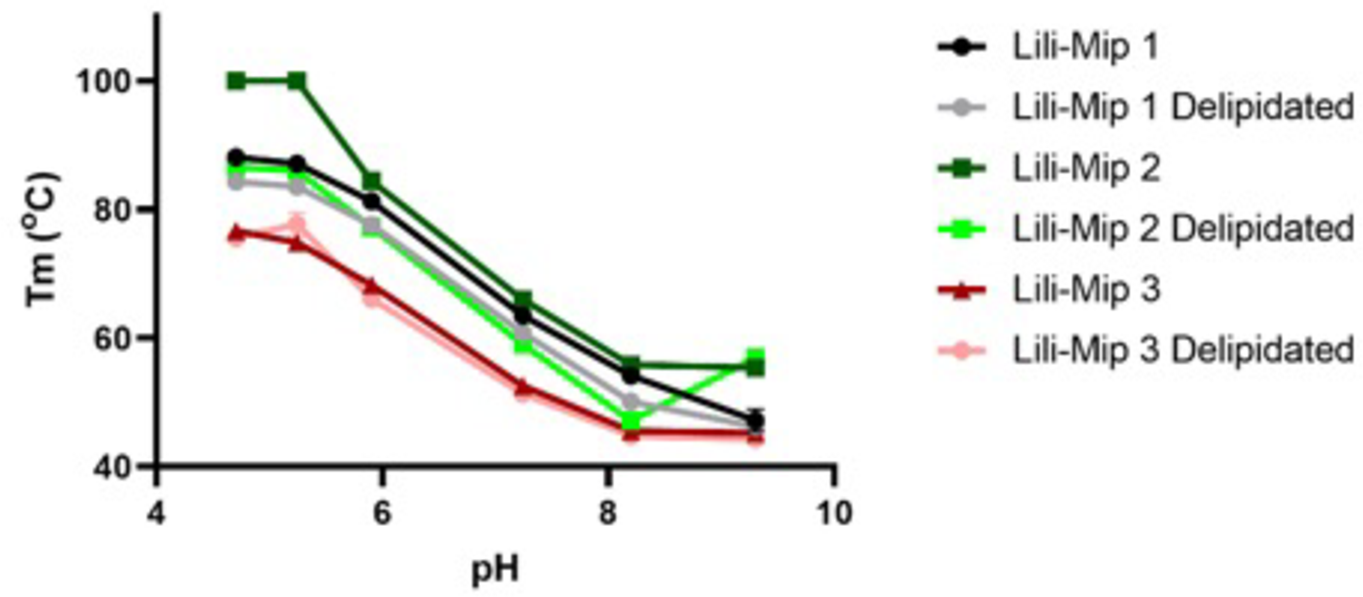

Supplement: S3 Fig — Thermal denaturation of Lili-Mip-1, 2, and 3 and delipidated ones show a marginal decrease in stability, more pronounced in acidic pH in Tycho. The experiments are repeated with three biological replicates, n = 3, and are drawn as mean ± S.D. (TIF) [file pone.0280009.s003.tif]

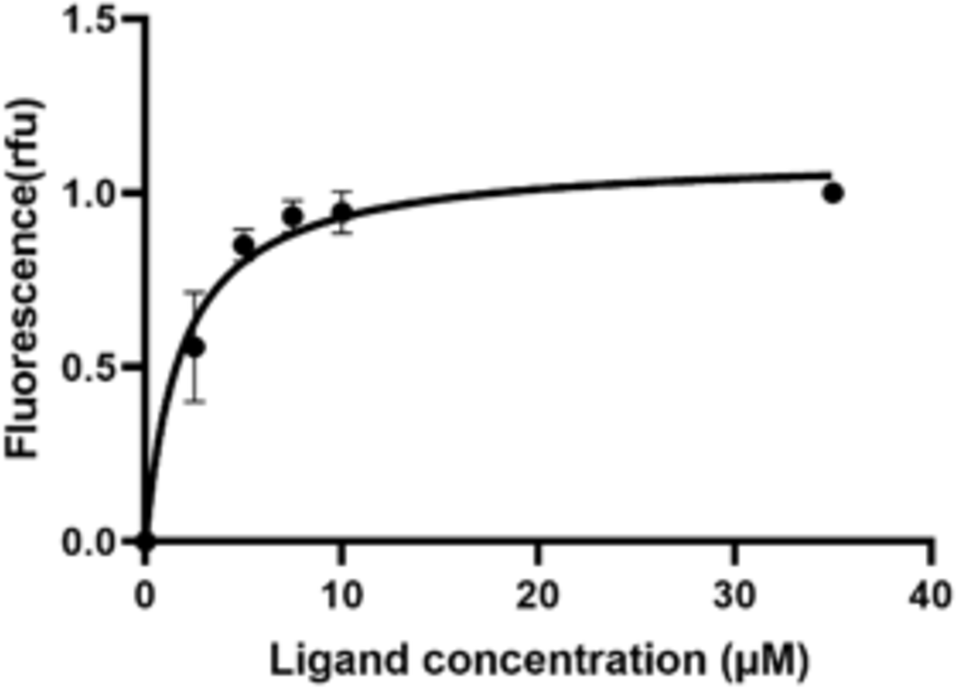

Supplement: S4 Fig — Representative figure showing the titration of Lili-Mip-1 with 0μM to 35μM myristic acid. The experiments are repeated three times (three biological replicates). (TIF) [file pone.0280009.s004.tif]

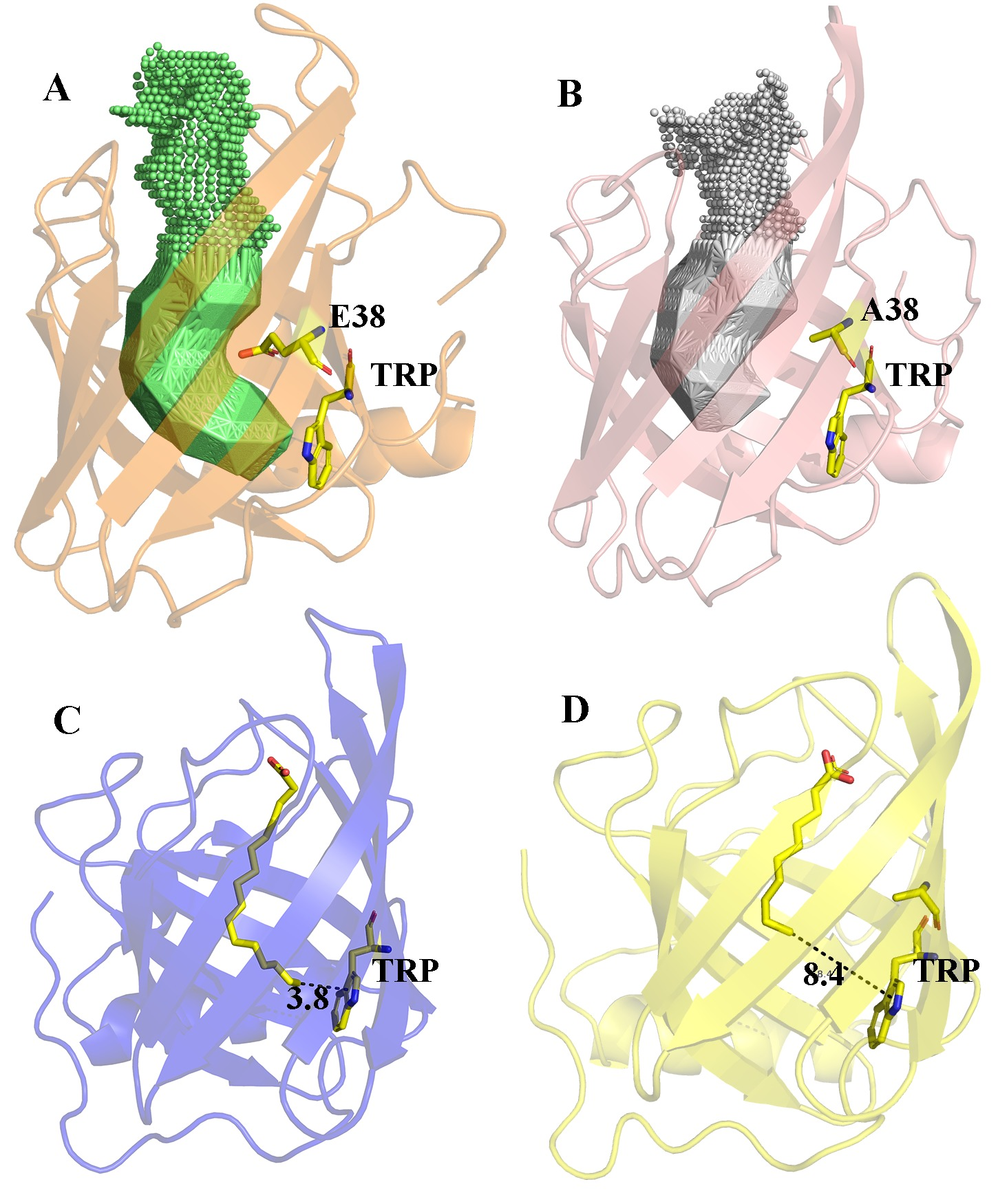

Supplement: S5 Fig — (A) shows the cavity of Lili-Mip2 in green. E38, as in the main paper Fig 1, shows a kink. E38 and the Tryptophan residue are marked. (B) The cavity of LiliMip 2 with E38A mutation–shows that the cavity does not extend close to the TRP. (C) The bound fatty acid in LiliMip 2 –the distance between the terminal methyl group and the indole ring of the tryptophan is 3.8Å. (D) The bound fatty acid in LiliMip 2 with E38 mutated to A. The distance between the terminal methyl group and the indole ring of tryptophan is 8.4Å. Cavity figures were made with the software CavityPlus [42] (TIF) [file pone.0280009.s005.tif]

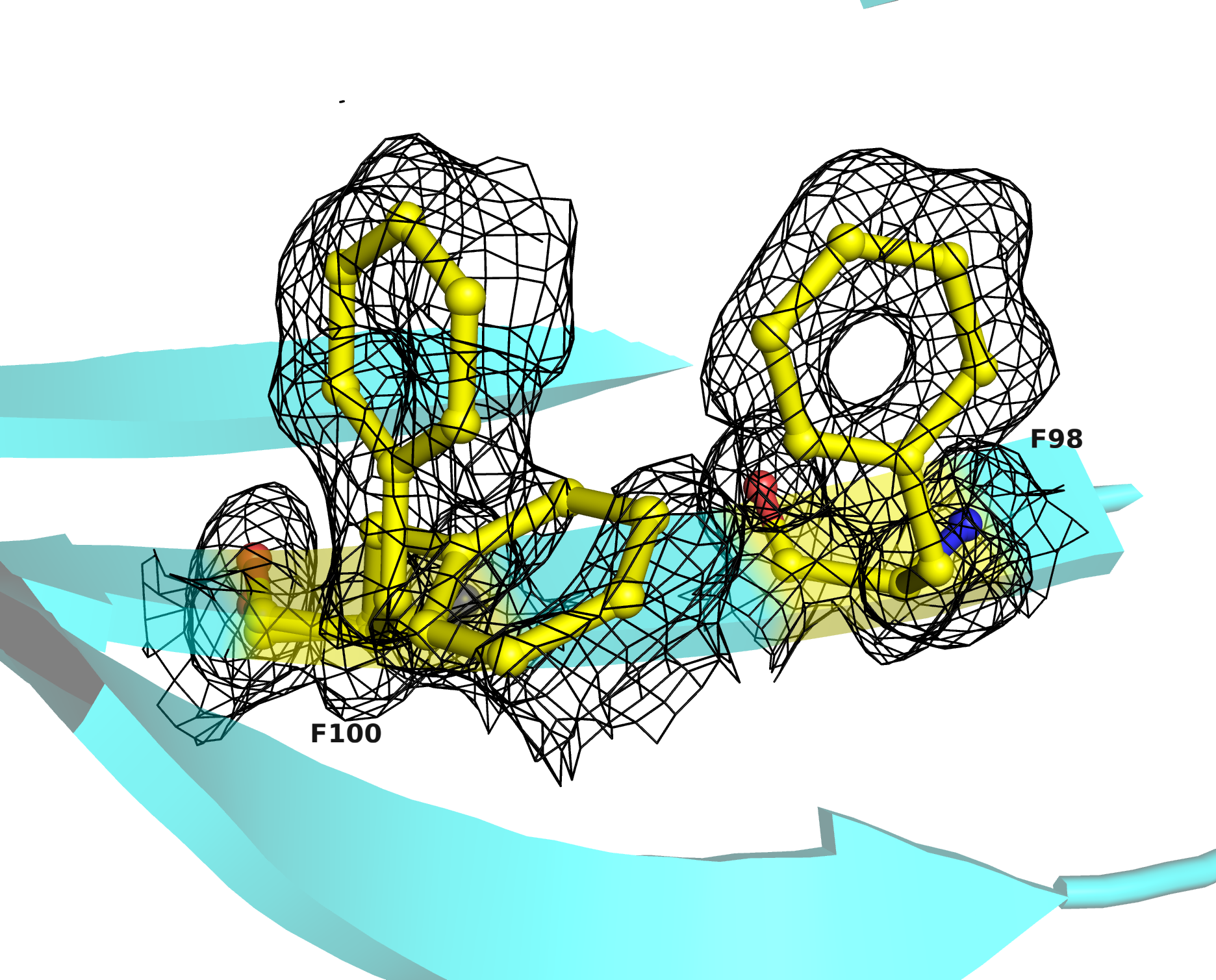

Supplement: S6 Fig — This demonstrates the flexibility of the side chain. The diagram was made by downloading the 4NYQ coordinates and 2Fo-Fc maps from the protein data bank. (TIF) [file pone.0280009.s006.tif]

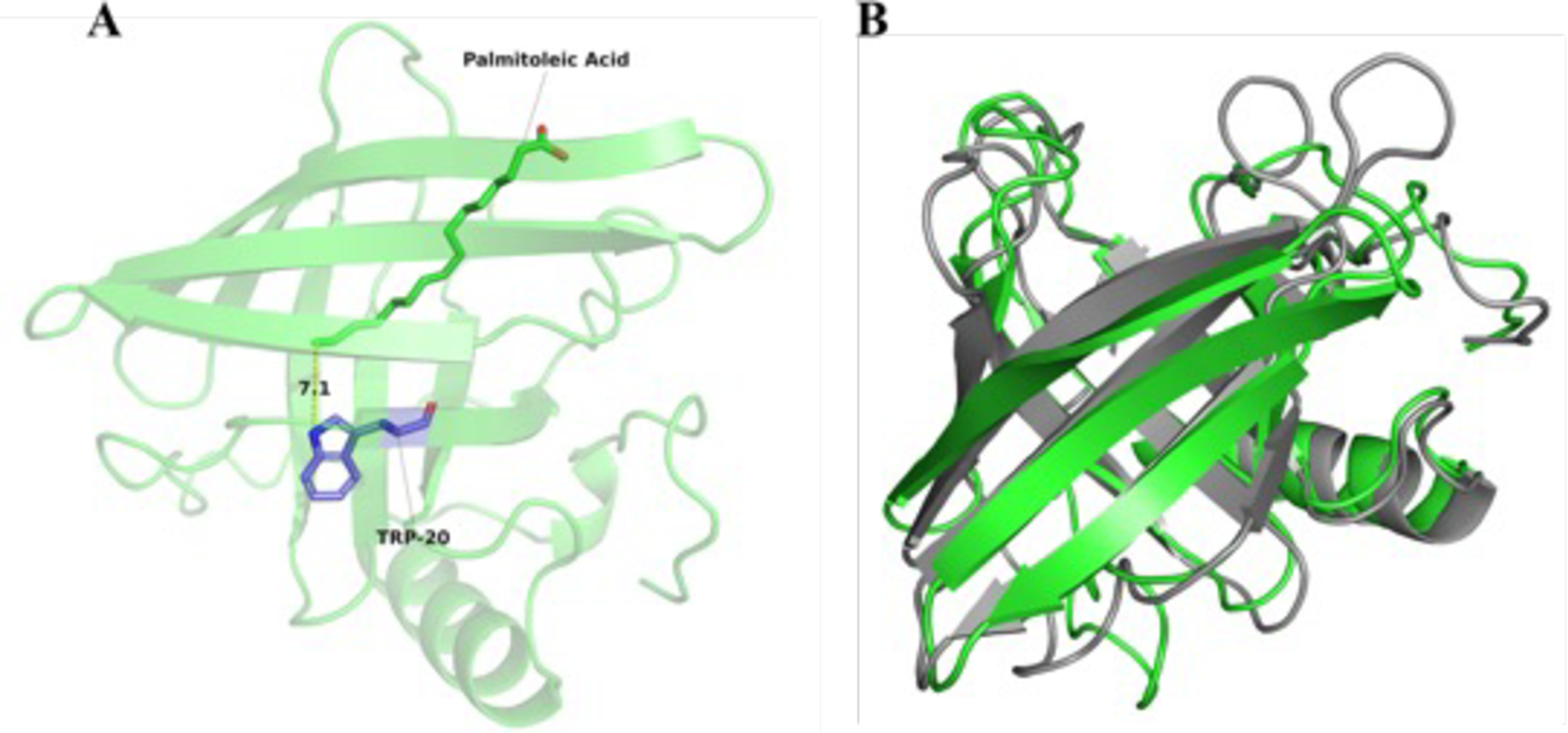

Supplement: S7 Fig — (A) Distance of W20 on LiliMip 2 and C16:1 on palmitoleic acid of docked conformation. (B) The final conformation of LiliMip 2 (green) from the MD simulation is superimposed on its crystal structure (grey). (TIF) [file pone.0280009.s007.tif]
