# Supplementary material for: Variability in phenylalanine side chain conformations facilitates broad substrate tolerance of fatty acid binding in cockroach milk proteins
Source: PLoS One. 2023 Jun 29;18(6):e0280009. doi: 10.1371/journal.pone.0280009 (PMC10310036; doi:10.1371/journal.pone.0280009)
Supplement: S1 Table — (DOCX) [file pone.0280009.s008.docx]

| Data collections | Lili-Mip-2-E38A | Lili-Mip-1 |
| --- | --- | --- |
| PDB ID | 8F0V | 8F0Y |
| Beamline | ALS 4.2.2 | APS 23-ID-D |
| Wavelength(Å) | 1.07 | 1.03 |
| Space Group | P22_1_2_1_ | P1 |
| Cell Parameters (Å) | 39.7 52.4 136.4, 90,90,90 | 32.7 33.0 38.6, 100.8, 99.7,103.6 |
| Resolution(Å) | 38.2 – 2.95 (2.94-2.62) | 37.0 – 2.1 (2.16-2.10) |
| R_meas_(%) | 0.140(2.214) | 0.175 (0.968) |
| R_pim_(%) | 0.054(0.837) | 0.107 (0.599) |
| *I/σ* | 11.5(1.0) | 3.5 (1.6) |
| CC(1/2) | 0.99 (0.57) | 0.98 (0.48) |
| Completeness(%) | 99.9 (100.0) | 96.7 (95.4) |
| Unique Reflections | 6461 | 8498(704) |
| Multiplicity | 6.6 | 2.6 |
| **Refinement** |  |  |
| R_work_/R_free_ (%) | 0.234/0.294 | 0.196/0.252 |
| RMS deviations: |  |  |
| Bond lengths(Å) | 0.01 | 0.015 |
| Bond angles(º) | 1.36 | 2.5 |
| Ramachandran(%) |  |  |
| Favored | 87 | 97 |
| Allowed | 11 | 3 |
| Outlier | 2 | 0 |

**Supplementary Table 1.** Data collection and refinement statistics for Lili-Mip-2-E38A and Lili-Mip-1
